# Spectroscopic Studies of Amino Acid Ionic Liquid-Supported Schiff Bases

**DOI:** 10.3390/molecules18054986

**Published:** 2013-04-29

**Authors:** Paula Ossowicz, Ewa Janus, Grzegorz Schroeder, Zbigniew Rozwadowski

**Affiliations:** 1 Department of Inorganic and Analytical Chemistry, West Pomeranian University of Technology, Szczecin, Al. Piastów 42, Szczecin 71-065, Poland; E-Mail: paulaossowicz@o2.pl; 2 Department of Organic Chemical Technology, West Pomeranian University of Technology, Szczecin, ul. Pułaskiego 10, Szczecin 70-332, Poland; E-Mail: ejanus@zut.edu.pl; 3 Faculty of Chemistry, A. Mickiewicz University, ul. Umultowska 89b, Poznań 61-614, Poland; E-Mail: schroede@amu.edu.pl

**Keywords:** amino acid ionic liquids, Schiff bases, deuterium isotope effect, intramolecular hydrogen bond

## Abstract

Amino acid ionic liquid-supported Schiff bases, derivatives of salicylaldehyde and various amino acids (l-threonine, l-valine, l-leucine, l-isoleucine and l-histidine) have been investigated by means of various spectroscopic techniques (NMR, UV-Vis, IR, MS) and deuterium isotope effects on ^13^C-NMR chemical shifts. The results have shown that in all studied amino acid ionic liquid-supported Schiff bases (except the l-histidine derivative) a proton transfer equilibrium exists and the presence of the COO^−^ group stabilizes the proton transferred NH-form.

## 1. Introduction

Ionic liquids (ILs) which are composed solely of ions [1] and are liquid at ambient temperature [2] have been widely studied as replacements for volatile organic solvents in various reactions. A particularly attractive advantage of ionic liquids is their negligible vapor pressure, which results in reduced air emissions, non-flammability and non-explosiveness [1,2,3]. In addition, other physical properties of ILs such as their polarity, hydrophobicity, hydrogen-bond basicity, viscosity and solvation interactions with organic and inorganic compounds can be carefully modulated through the proper selection of cations and anions [4,5,6,7,8]. Recently, much attention has been paid to a new generation of ionic liquids originating from natural raw materials such as amino acids. Amino acid ionic liquids are an alternative to traditional ionic liquids, based solely on petrochemical raw materials. Amino Acid Ionic Liquids (AAILs) derived from biorenewable raw materials, have increased biocompatibility of the ionic liquid, expressed by greater ability to biodegrade in the environment, and also have lower toxicity (both ecotoxicity and cytotoxicity) [9,10].

Organic salts of amino acid Schiff bases can be excellent candidates for ionic liquids because of a large selection of aldehydes, amino acids, presence or lack of intramolecular hydrogen bonds as well as various cations which can strongly influence the acquired properties of ionic liquids [11,12]. It is well known that Schiff bases derived from amino acids and *ortho*-hydroxy aldehydes show biological activity and are widely applied as ligands of complexes used as enantioselective catalysts. In various biologically important reactions, Schiff bases are formed as intermediate products [13]. The presence of intramolecular hydrogen bond is essential for their enzymatic function and the proton transfer process from oxygen to nitrogen atom is the first step of the catalytic cycle [14].

Moreover, the possibility of use of the amino acid ionic liquid-supported Schiff bases as the chiral solvents as well as ligands of the catalysts makes this class of compounds especially promising. Ionic liquid-supported Schiff bases, derivatives of 1-(2-aminoethyl)-3-methylimidazolium hexafluorophosphate and aromatic aldehydes, were investigated as ligands and as solvents for the Pd-catalyzed Suzuki-Miyaura coupling reaction, with good to excellent yields of biaryls being obtained [15,16].

Most authors working on amino acid ionic liquids have been interested in their synthesis and some physical properties, e.g., polarity, thermal stability or miscibility. A new trend in ionic liquid research concerns more detailed studies of factors that can strongly affect their properties, e.g., interactions with solvents in Diels-Alder reactions. The use of NMR data has been proposed for prediction of the reaction selectivity [17,18].

**Figure 1:**
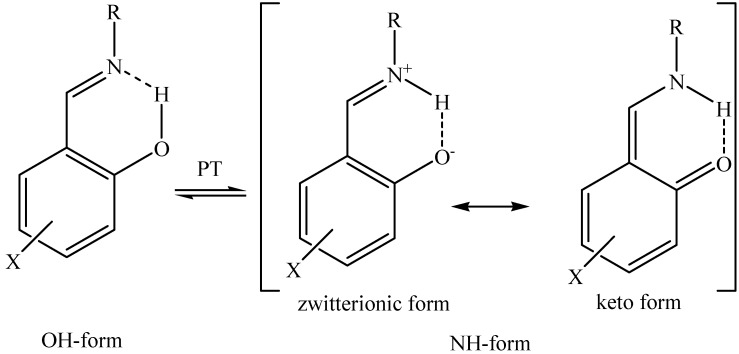
Proton transfer equilibrium in Schiff bases.

Previously we have studied the proton transfer equilibrium in chloroform solution of tetrabutylammonium salts of amino acid Schiff bases, derivatives of 2-hydroxynaphthaldehyde [19] and lithium salts of these Schiff bases in DMSO [20] and water [21] by means of NMR spectroscopy. In this paper we extend our studies to other techniques (IR, UV-Vis, MS) and investigate derivatives of salicylaldehyde, which can be considered as a new group of amino acid ionic liquids. We focus our attention on tetrabutylammonium salts of Schiff base derivatives of salicylaldehyde and different amino acids. Proton transfer equilibria (Figure 1) have been also studied because they are crucial for investigation of their properties. Organic salts of amino acid Schiff bases, derivatives of 2-hydroxyaldehyde and substituted salicylaldehydes exist mainly in pure proton transferred form [19]. Selection of the amino acids (l-threonine, l-leucine, l-isoleucine, l-histidine) and salicylaldehyde can influence the position of the proton in intramolecular hydrogen bonds and hence can allow us to obtain amino acid ionic liquids with the desired required position of the proton transfer equilibrium.

In proton transfer equilibrium studies, the measurements of deuterium isotope effects on chemical shift are especially effective. This method allows detection of the presence of proton transfer equilibria and determination of the respective mole fractions of tautomers. Measurements of deuterium isotope effects on chemical shifts can be performed as a one tube experiment for a partially deuterated sample. The position of the proton transfer equilibrium in Schiff bases was determined on the basis of deuterium isotope effects on ^13^C-NMR chemical shifts [22]. The deuterium isotope effects were measured as differences between the ^13^C signals in the spectra of non- deuterated and deuterated species: ^n^ΔC(D) = δC(H) − δC(D).

## 2. Results and Discussion

### 2.1. NMR, IR and UV-Vis

**Figure 2:**
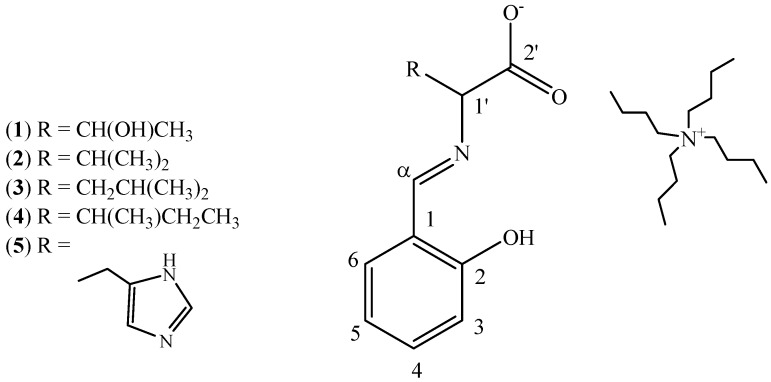
Amino acid ionic liquids supported Schiff bases **1**–**5**.

The ^1^H- and ^13^C-NMR chemical shifts for all positions and the whole range of temperatures (295, 270, 250 and 230 K) for compounds **1**–**5** (Figure 2) in CDCl_3_ and DMSO solutions are given in Table 1. Some of the data for compound **3** in CDCl_3_ have been taken from [19]. ^1^H chemical shifts in CDCl_3_ for compound **3** at position H-3 and H-6 were corrected to those from reference [19].

**Table 1:** ^1^H and ^13^C chemical shifts (ppm), deuterium isotope effects (ppb) and ^3^*J*_(NH,H)_ coupling constants (Hz) of compounds **1**–**5** in CDCl_3_ and DMSO.

Comp.	T (K)			Position										^3^*J*_(NH.H)_
				1	2	3	4	5	6	α	2'	1'	Others	
(1) Sal-L-Thr	295	CDCl_3_	δH (ppm)	-	**14.41**	6.85	~7.25	6.78	~7.25	**8.45**	**-**	4.16	3.92;3.18;1.56;1.37;1.20;0.95	n.o.
			δC (ppm)	119.01	**165.20**	117.46	132.06	117.59	131.50	**163.14**	**173.97**	76.09	68.13;58.54;23.86;19.67;19.11;13.65	
			ΔC(D) (ppb)	n.o.	**136**	n.o.	n.o.	n.o.	n.o.	**451**	**n.o.**	−174		
	270	CDCl_3_	δH (ppm)	-	**14.49**	6.85	~7.3	6.80	~7.3	**8.46**	**-**	4.17	3.94;3.16;1.54;1.36;1.21;0.95	n.o.
			δC (ppm)	118.81	**165.27**	117.36	132.12	117.69	131.48	**162.85**	**174.07**	75.93	68.00;58.23;23.67;19.59;19.05;13.69	
			ΔC(D) (ppb)	−155	**174**	-88	57	152	−72	**527**	**77**	−178		
	250	CDCl_3_	δH (ppm)	-	**14.55**	6.87	~7.3	6.83	~7.3	**8.46**	**-**	4.18	3.96;3.16;1.53;1.34;1.23;0.96	n.o.
			δC (ppm)	118.65	**165.36**	117.30	132.20	117.79	131.49	**162.62**	**174.18**	75.78	67.91;58.00;23.52;19.54;19.01;13.75	
			ΔC(D) (ppb)	−145	**174**	−86	67	139	−64	**535**	**74**	−178		
	230	CDCl_3_	δH (ppm)	-	**14.62**	6.89	7.36	6.85	7.31	**8.47**	**-**	4.18	3.99;3.14;1.53;1.33;1.23;0.96	n.o.
			δC (ppm)	118.48	**165.41**	117.26	132.30	117.88	131.49	**162.41**	**174.28**	75.57	67.81;57.77;23.38;19.50;18.97;13.81	
			ΔC(D) (ppb)	136	**174**	−86	68	149	−73	**540**	**83**	−235		
	295	DMSO	δH (ppm)	-	**14.32**	6.76	7.24	6.74	7.34	**8.37**	**-**	3.81	3.60;3.15;1.55;1.30;1.02;0.92	n.o.
			δC (ppm)	118.26	**164.49**	116.65	132.06	117.40	131.51	**163.68**	**170.95**	75.10	67.35;57.38;22.95;19.44;19.11;13.40	
(2)Sal-L-Val ^a^	295	CDCl_3_	δH (ppm)	-	**14.75**	6.79	7.23	6.66	7.17	**8.21**	**-**	3.71	3.24;1.57;1.35;1.00;0.94	n.o.
			δC (ppm)	117.85	**167.22**	119.11	132.89	115.95	131.85	**163.39**	**173.44**	79.11	58.57;31.45;23.94;19.70;18.34;13.67	
			ΔC(D) (ppb)	n.o.	**n.o.**	n.o.	n.o.	n.o.	n.o.	**n.o.**	**n.o.**	n.o.		
	270^b^	CDCl_3_	δH (ppm)	-	**14.90**	6.79	7.24	6.65	7.18	**8.17**	**-**	3.73	3.24;1.57;1.35;0.99;0.94	br.
			δC (ppm)	117.38	**168.07**	119.46	133.21	115.61	131.93	**163.34**	**173.28**	78.58	58.28;31.32;23.77;19.62;18.14;13.72	
			ΔC(D) (ppb)	n.o.	**74 ^c^**	n.o.	−48	−51	−135	**269**	**n.o.**	n.o.		
	250	CDCl_3_	δH (ppm)	-	**14.89**	6.80	7.28	6.67	7.20	**8.16**	**-**	3.76	3.20;1.55;1.33;1.00;0.93	6.1
			δC (ppm)	117.07	**168.38**	119.58	133.46	115.55	132.04	**163.51**	**173.43**	78.06	58.00;31.21;23.58;19.56;18.45;17.65;13.83	
			ΔC(D) (ppb)	29	**−73**	n.o.	−75	−105	−156	**271**	**n.o.**	n.o.		
	230	CDCl_3_	δH (ppm)	-	**15.00**	6.80	7.31	6.67	7.22	**8.14**	**-**	3.81	3.20;1.54;1.31;1.00;0.94	7.1
			δC (ppm)	116.61	**169.66**	120.09	133.91	115.19	132.18	**163.52**	**173.35**	~77.4	57.78;31.09;23.43;19.52;18.45;17.65;13.83	
			ΔC(D) (ppb)	80	**br.**	br.	−183	−149	−118	**283**	**n.o.**	n.o.		
	295	DMSO	δH (ppm)	-	**14.54**	6.68	7.29	6.62	7.21	**8.28**	**-**	3.44	3.15;2.26;1.55;1.29;1.04;0.92;0.84;0.82	n.o.
			δC (ppm)	117.68	**166.36**	118.49	132.56	115.55	131.85	**163.35**	**170.63**	77.98	57.50;56.01;30.86;23.08;20.27;19.23;18.58;18.11;13.52	
(3)Sal-L-Leu	295	CDCl_3_	δH (ppm)	-	**14.76**	6.79	7.22	6.68	7.18	**8.32**	**-**	4.02	3.23;1.91;1.68;1.57;1.37;0.96;0.92;0.89	n.o.
			δC (ppm)	118.20	**166.11**	118.73	132.60	116.34	131.84	**163.14**	**174.21**	71.25	58.59;43.09;25.03;23.93;21.50;19.71;18.49;13.67	
			ΔC(D) (ppb)	n.o.	**n.o.**	n.o.	n.o.	n.o.	n.o.	**n.o.**	**n.o.**	n.o.		
	270	CDCl_3_	δH (ppm)	-	**14.83**	6.80	7.25	6.69	7.20	**8.30**	**-**	4.03	3.20;1.91;1.63;1.56;1.35;0.95;0.91;0.88	n.o.
			δC (ppm)	117.83	**166.36**	118.85	132.83	116.25	131.90	**163.22**	**174.30**	70.85		
			ΔC(D) (ppb)	n.o.	**248**	n.o.	n.o.	-47	−103	**248**	**n.o.**	-113	58.24;42.77;24.83;23.71;21.18;19.63;18.50;13.73	
	250	CDCl_3_	δH (ppm)	-	**14.84**	6.81	~7.3	6.71	7.22	**8.30**	**-**	4.05	3.17;1.92;1.54;1.32;0.95;0.88	3.3
			δC (ppm)	117.20	**166.87**	119.00	133.04	116.10	131.96	**163.56**	**174.37**	70.35	58.00;42.51;24.66;23.71;20.92;19.57;18.46;13.77	
			ΔC(D) (ppb)	n.o.	**198**	n.o.	n.o.	−44	−121	**259**	**n.o.**	-104		
	230	CDCl_3_	δH (ppm)	-	**14.95**	6.82	7.30	6.72	7.24	**8.27**	**-**	4.06	3.18;1.94;1.54;1.33;0.95;0.91;0.87	7.3
			δC (ppm)	117.19	**167.24**	119.23	133.31	116.03	131.93	**163.68**	**174.45**	69.88	57.78;42.24;24.51;23.76;20.64;19.53;13.84	
			ΔC(D) (ppb)	n.o.	**146**	n.o.	n.o.	n.o.	869	**256**	**n.o.**	-76		
	350	DMSO	δH (ppm)	-	**14.20**	6.69	7.19	6.64	7.26	**8.35**	**-**	3.72	3.20;1.78;1.61;1.34;0.94;0.88;0.86	n.o.
			δC (ppm)	118.59	**166.95**	118.64	132.52	116.12	132.0	**162.95**	**171.26**	71.13	58.53;43.89;25.37;23.75;22.44;19.69;13.73	
	330		δH (ppm)	-	**14.4**	6.69	7.20	6.64	7.28	**8.35**	**-**	3.70	3.19;1.75;1.61;1.32;0.93;0.88;0.86	
			δC (ppm)	118.51	**166.14**	118.62	132.61	116.17	132.06	**163.07**	**171.39**	71.10	58.31;43.79;25.31;23.74;22.35;19.68;13.82	
	295		δH (ppm)	-	**14.53**	6.68	7.28	6.63	7.20	**8.33**	**-**	3.66	3.16;1.90;1.55;1.29;0.92;0.86;0.83	
			δC (ppm)	117.70	**165.73**	118.15	132.25	115.59	131.63	**162.69**	**170.81**	70.33	57.37;43.01;24.61;23.28;22.95;21.55;19.10;13.39	
(4)Sal-L-Ile	295	CDCl_3_	δH (ppm)	-	**14.81**	6.79	7.23	6.64	7.16	**8.18**	**-**	3.74	3.23;2.87;2.24;1.66;1.56;1.35;1.23;0.97;0.93;0.88	n.o.
			δC (ppm)	117.77	**167.56**	119.31	133.01	115.86	131.90	**163.18**	**173.56**	78.74	58.60;37.95;24.98;23.95;19.72;16.51;13.67;11.56	
			ΔC(D) (ppb)	n.o.	**n.o.**	n.o.	n.o.	n.o.	n.o.	**n.o.**	**n.o.**	n.o.		
	270	CDCl_3_	δH (ppm)	-	**14.89**	6.78	7.24	6.65	7.18	**8.16**	**-**	3.78	3.23;2.27;1.68;1.56;1.33;1.23;1.16;0.96;0.91;0.88	br.
			δC (ppm)	117.31	**168.24**	119.55	133.30	115.60	131.96	**163.24**	**173.52**	78.28	58.24;37.75;24.69;23.73;19.63;16.44;13.72;11.61	
			ΔC(D) (ppb)	n.o.	**−43 ^c^**	−58	35	n.o.	−145	**254**	**n.o.**	n.o.		
	250	CDCl_3_	δH (ppm)	-	**14.94**	6.79	7.27	6.66	7.19	**8.14**	**-**	3.80	3.21;2.28;1.68;1.56;1.30;1.23;1.16;0.95;0.90;0.88	6.0
			δC (ppm)	116.94	**168.78**	119.78	133.60	115.42	132.05	**163.30**	**173.57**	77.87	58.01;37.63;24.44;23.57;19.57;16.38;13.77;11.67	
			ΔC(D) (ppb)	59	**−41**	−112	-83	n.o.	−164	**276**	**n.o.**	101		
	230	CDCl_3_	δH (ppm)	-	**15.03**	6.79	7.31	6.64	7.21	**8.10**	**-**	3.85	3.22;2.33;1.70;1.55;1.32;1.23;1.17;0.95;0.90;0.88	7.2
			δC (ppm)	116.46	**169.85**	120.24	134.02	115.03	132.22	**163.37**	**173.46**	77.41	57.78;37.49;24.15;23.43;19.54;16.35;13.83;11.76	
			ΔC(D) (ppb)	85	**-143**	−150	−134	95	−158	**295**	**n.o.**	ov.		
	295	DMSO	δH (ppm)	-	**14.57**	6.66	7.27	6.60	7.20	**8.26**	**-**	3.44	3.15;1.99;1.50;1.29;1.00;0.93;0.83	
			δC (ppm)	117.56	**166.83**	118.67	132.58	115.27	131.86	**163.05**	**170.24**	77.37	57.49;37.56;24.63;23.06;19.22;16.60;13.52;11.67	
(5) Sal-L-His	295	CDCl_3_	δH (ppm)	-	**12.33**	6.63	~7.3	6.80	~7.3	**7.53**	**-**	5.19	3.75;3.58;3.32;3.14;2.94;1.54;1.30;0.92	
			δC (ppm)	~123.7	**~157.3**	116.22	~128.3	118.88	~129.6	**n.o.**	**n.o.**	58.67	58.31;~26.45;23.93;19.69;13.67	
			ΔC(D) (ppb)	n.o.	**n.o.**	n.o.	n.o.	n.o.	n.o.	**n.o.**	**n.o.**	n.o.		
	270	CDCl_3_	δH (ppm)	-	**12.75**	6.63	7.08	6.81	~7.3	**7.47**	**-**	5.20	11.82;3.77;3.36;3.09;2.98;1.52;1.26;0.91	
			δC (ppm)	123.71	**157.11**	116.10	126.47	118.88	129.46	**135.53**	**175.35**	59.81	58.31;27.34;23.70;19.57;13.69	
			ΔC(D) (ppb)	n.o.	**n.o.**	n.o.	n.o.	n.o.	n.o.	**n.o.**	**n.o.**	n.o.		
	250	CDCl_3_	δH (ppm)	-	**12.91**	6.64	7.09	6.83	~7.3	**7.44**	**-**	5.19	11.87;3.80;3.65;3.40;3.07;~3.0;1.51;1.25;0.91	
			δC (ppm)	123.74	**156.87**	116.05	126.21	118.89	129.47	**135.10**	**175.60**	59.64	134.87;58.05;27.15;23.55;19.49;13.72	
			ΔC(D) (ppb)	157	**n.o.**	n.o.	n.o.	n.o.	136 ^a^	**393**	**n.o.**	-88 ^a^		
	230	CDCl_3_	δH (ppm)	-	**13.06**	6.65	7.11	6.85	~7.3	**7.47**	**-**	5.20	11.91;3.83;3.67;3.43;3.05;1.51;1.25;0.91	
			δC (ppm)	123.58	**156.82**	115.98	126.16	118.88	129.38	**135.01**	**175.69**	59.49	128.24;57.26;26.94;23.44;19.45;13.77	
			ΔC(D) (ppb)	143	**br.**	-41 ^c^	-28 ^c^	64	br.	**br.**	**n.o.**	br.	40	
	295	DMSO	δH (ppm)	-	**12.1**	6.61	7.06	6.74	7.19	**7.30**	**-**	5.04	~11.6;3.19;3.14;~2.9;1.57;1.33;0.96	
			δC (ppm)	124.47	**157.79**	116.41	127.80	118.38	129.85	**133.85**	**172.83**	58.91	136.09;129.85;58.0;26.72;23.57;19.72; 14.00	

n.o. not observed; br. broad signals; ^a^ Data in CDCl_3_ at 295, 250 and 230K from reference [19]; ^b^ Data from partly deuterated compound; ^c^ Uncertain value.

The type of amino acids group usually has a small effect on the positions of the ^13^C signals assigned to the phenyl ring. The C-2 chemical shift values are the most sensitive to the position of the hydrogen engaged in the intramolecular hydrogen bond and vary in CDCl_3_ solution at 295K from δ 165.2 for the l-treonine derivative up to δ 167.6 ppm for that of l-isoleucine. Moreover it increases as the temperature is lowered. The greatest change of up to D = 2.19 ppm, was observed for compound **3**, and the lowest D = 0.19 ppm for the l-threonine derivative (**1**). Similar effects were observed for other salts of amino acid Schiff bases [19,20,21]. The type of amino acid had a small influence of the chemical shift of the C=O carbon. The chemical shift values of the C=O carbon in the compounds studied were similar to those determined for other Schiff bases involving R-substituted salicylaldehydes [19]. For the l-histidine derivative **5** a shielding effect of the imidazole ring was observed for H-α and C-α. The difference in the chemical shifts of signals H-α and C-α for compounds **1**–**4** and compound **5** (the l-histidine derivative) is 1 ppm for H-α and about 30 ppm for C-α.

**Figure 3:**
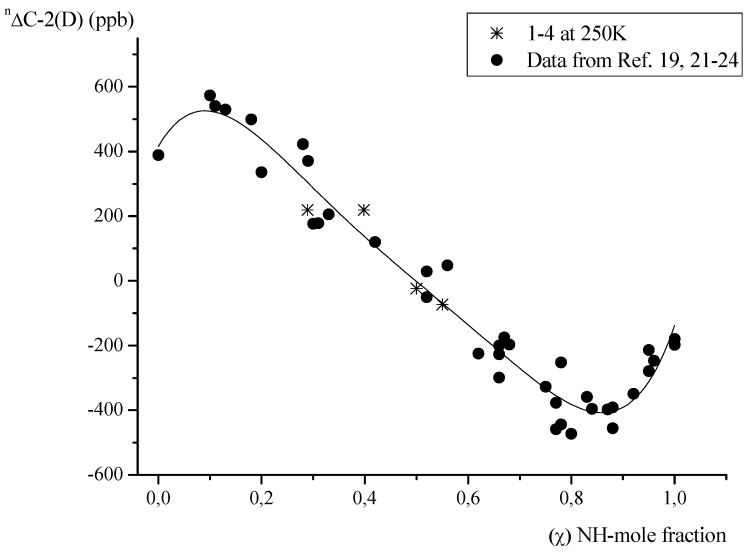
Plot of DC-2(D) *vs.* mole fraction of the proton transferred NH-form (χ).

All observed deuterium isotope effects are presented in Table 1. Large positive values of deuterium isotope effects in the range from *ca.* 250 up to *ca.* 550 ppb were found for C-α. Negative values were observed for C-6. Both positive and negative values were observed for the other carbons. It is known that the value of deuterium isotope effect on the carbon atom linked to the phenolic group reflects the position of proton in the hydrogen bridge [22,23]. On the basis of the DC-2(D) *vs.* mole fraction of the NH-form relationship, it is possible to estimate the position of the equilibrium (Figure 3) in Schiff bases.

**Figure 4:**
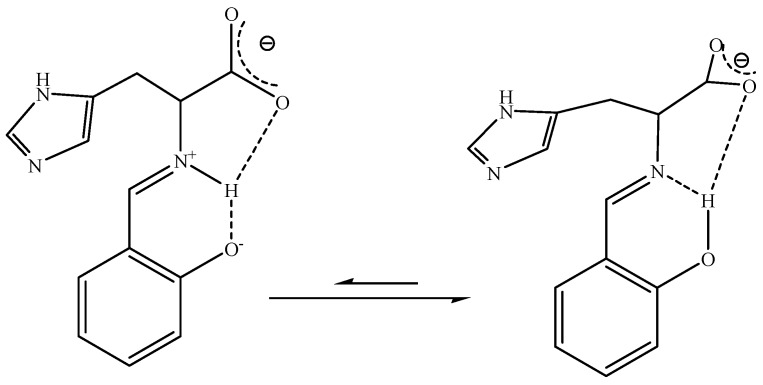
Influence of the imidazole ring on the conformation of **5**.

Values of DC-2(D) which are the most sensitive to the position of proton in the hydrogen bridge, are in the range from ~250 to −75 ppb, so close to those observed for other derivatives of substituted salicylaldehydes in which the proton transfer equilibrium exists [24]. For the derivatives of l-valine, l-leucine and l-isoleucine at 230K the mole fractions of NH-form estimated from Figure 3, were close to 0.4–0.5. The values of DC-2(D) observed for the l-threonine derivative indicated the presence of an equilibrium shifted towards the OH-form (χ equals *ca.* 0.3). ^3^*J*(NH,H) coupling constants as well as δC-2 chemical shift values (Table 1) confirmed the equilibrium positions for compounds **1**–**4**. A lower mole fraction of the NH-form for the l-threonine derivative and its small temperature dependence can be related to the presence of hydroxyl groups in the amino acid chain. A similar situation was observed for a Schiff base derivative of 2-hydroxynaphthaldehyde and l-threonine in which the proton transfer equilibrium was shifted towards the OH-form [25], while the derivatives of other amino acids exist almost exclusively in the NH-form [20,21]. Although several attempts have been taken to measure a DC-2(D) value for compound **5**, it was not possible due to the broad and low intensity signal of the C-2 carbon, even at low temperatures. However, on the basis of the DC-2(D) *vs.* DC-1'(D) relationship found for Schiff base derivatives of various salicylaldehydes and aliphatic amines [24] the DC-1'(D) value of *ca.* -90 ppb suggests that DC-2(D) is close to ~400 ppb, which is typical of the OH-form. The position of the δC-2 signal at *ca.* 157 ppm for the l-histidine derivative **5** is also typical of Schiff bases in which a proton transfer equilibrium does not exist [24]. Comparison of the chemical shifts of carbon C-2, DC-2(D) as well as ^3^*J*(NH,H) coupling constants in CDCl_3_ for the other studied Schiff bases **1**–**4** has also indicated that the presence of the l-histidine group shifted the position of the proton transfer equilibrium towards the pure OH-form in **5**. This shift of the equilibrium reveals that the conformation forced by the position of imidazole ring of l-histidine weakens the interactions between the COO- and NH^+^ groups which stabilize the proton transferred form (Figure 4).

A similar situation was observed for Schiff base derivatives of l-phenylalanine and salicylaldehyde, in which the mole fraction of the NH-form was close to 0.2 at 230K [19]. However, for **5** this effect is particularly large. For compound **5** the δC-2 values in range *ca.* 155–157 ppm are typical of the pure OH-form [24] while for other tetrabutylammonium salts of l-phenylalanine and various salicylaldehydes these values were in the 165–170 ppm range [19].

Deuterium isotope effects observed on the carboxylic carbon C-2' for compounds **1** and **4** (*ca.* 80 ppb) can be related to engagement of the COO^−^ group in hydrogen bond [19].

The chemical shift values in DMSO solution of the C-2' carbons for the compounds studied (δ 170–171 ppm) are close to those observed for lithium salts of amino acid Schiff base derivatives of 2-hydroxynaphthaldehyde, and indicate that the counter-ion is in the proximity of the carboxylic acid group. The δC-2' values found in chloroform solution (δ173–176 ppm) are closer to those observed for lithium salts of amino acid Schiff bases in D_2_O (178–180ppm) [21]. DMSO has only a slight influence on the position of other signals. The influence of solvent on the position of equilibrium was small, judging by C-2 chemical shift values. Changes in chemical shifts related to the type of solvent used can be explained by small changes in the strength of the intermolecular hydrogen bond [20].

The length of the carbon chain of amino acid had a small influence on the position of the proton transfer equilibrium. For the l-valine, l-leucine and l-isoleucine derivatives the mole fractions of the NH-form are similar. Even for the l-threonine derivative the position of equilibrium is close to those estimated for compounds **2**–**3**. A similar situation was observed for the other Schiff base derivatives of amino acids and aromatic hydroxyaldehydes [19].

The characteristic IR absorption bands of amino acid ionic liquid-supported Schiff bases are presented in Table 2. In the IR spectra of the compounds studied the absorptions in the *ca.* 3500–2500 cm^−1^ range have been assigned to an intramolecularly bonded XH group. The *ν*(C=N) band occurs at ~1630 cm^−1^. The bands at ~1610 and ~1370 cm^−1^ are assigned to *ν*(COO^−^ sym.) and *ν*(COO^−^ asym.) [26,27].

**Table 2:** Selected IR brands of amino acids ionic liquid-supported Schiff bases **1**–**5** (cm^−1^).

Comp.	*v*(XH)	*v*(C=N)	*v*(COO^−^)as.	*v*(COO^−^)sym.
**(1) S-Thr**	3700–2700 br.	1630	1613	1377
**(2) S-Val**	3750–2700 br.	1630	1609	1368
**(3) S-Leu**	3700–2700 br.	1629	1606	1361
**(4) S-Ile**	3700–2875 br.	1629	1608	1361
**(5) S-His**	3500–2400 br.	1597	1597 ov.	1391

UV–Vis measurements were also performed for compounds **1**–**5**. The UV-Vis bands of the studied amino acids ionic liquids-supported Schiff bases in ethanol and chloroform are presented in Table 3.

**Table 3:** UV-Vis bands of compounds **1**–**5** in ethanol and chloroform solution (nm). In parentheses absorbance values.

Comp.	EtOH		CHCl_3_	
**(1) Sal-L-Thr**	288 (0.399)	404 (0.278)	292 (0.152)	419 (0.038)
	317 (0.447)		315 (0.138)	
**(2) Sal-L-Val**	289 (0.718)	404 (0.584)	288 (0.273)	408 (0.188)
	316 (0.699)		315 (0.2)	
**(3) Sal-L-Leu**	289 (0.596)	405 (0.429)	291 (0.478)	410 (0.315)
	315 (0.589)		315 (0.404)	
**(4) Sal-L-Ile**	288 (0.627)	407 (0.536)	289 (0.27)	408 (0.175)
	315 (0.572)		313 (0.185)	
**(5) Sal-L-His**	290 (1.218)	407 (0.137)	289 (0.926)	n.o.

The UV-Vis spectra of all compounds show a low energy band at ~410 nm and two high energy bands at ~288 and ~315 nm. On the basis of the results obtained for other derivatives of different aliphatic and aromatic amines these bands can be assigned to the OH-form, while the one at 410 nm corresponds to the NH form [21,27,28,29]. The UV-Vis results indicate that the compounds studied exist in an equilibrium of NH- and OH-forms, which is in agreement with the NMR results. For all compounds studied a more polar solvent shifted the proton transfer equilibrium towards the NH-form [27,28,29]. Even for compound **5** which in a chloroform solution is in the pure OH-form, in an ethanol solution a small amount of the NH-form was found. 

The specific and molar rotation values are summarized in Table 4. No correlation between the type of amino acid and the molar rotation was observed. Molar rotation was stable in time.

**Table 4:** Specific and molar rotation of amino acids ionic liquid-supported Schiff bases.

Comp.	[α]_λ_^T^	[M]_λ_^T^
**(1) Sal-L-Thr**	+13.6	+63.2
**(2) Sal-L-Val**	+29.7	+137.4
**(3) Sal-L-Leu**	+4.9	+23.4
**(4) Sal-L-Ile**	+17.7	+84.4
**(5) Sal-L-His**	+0.7	+50.1

### 2.2. ES MS

ESI mass spectra of negative and positive ions were recorded for compounds **1**–**5**. The positive region of ESI MS shows only one characteristic signal at *m/z* 242, assigned to tetrabutylammonium cations. Analysis of the ESI MS spectra of the compounds studied in the range of negative signals at low cone voltage confirmed the presence of the target compounds, evidenced by the *m/z* = M^−^ signal. Increasing cone voltage used in subsequent experiments led to strong fragmentation of the molecules. 

**Scheme 1:**
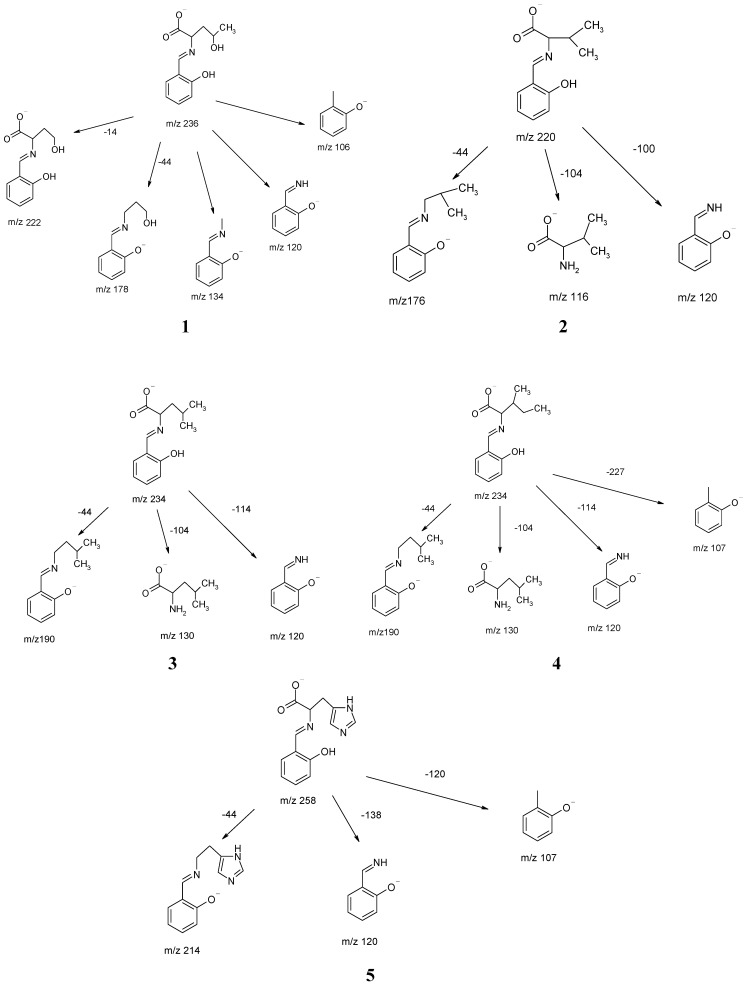
The ESI MS pathof fragmentation of compounds **1**–**5**.

The spectra recorded for negative ions as a function of the cone voltage not only determine the fragmentation pathway of anions, but also characterize the stability of ions in the gas phase. The fragment ions observed for Schiff base compounds **1**–**5** are given in Scheme 1.

For compounds **1**–**5** a few specific processes or ions were observed. The molecular ions as the precursor ionic species P^−^ eliminated a CO_2_ molecule in the decarboxylation reactions [30]. The characteristic signals at *m/z* 107 and 120 observed for the studied compounds appear as a result of elimination of the amino acid fragment. The specific fragments characteristic of various Schiff bases are determined by the structure of a given amino acid [30,31,32,33].

**Figure 5:**
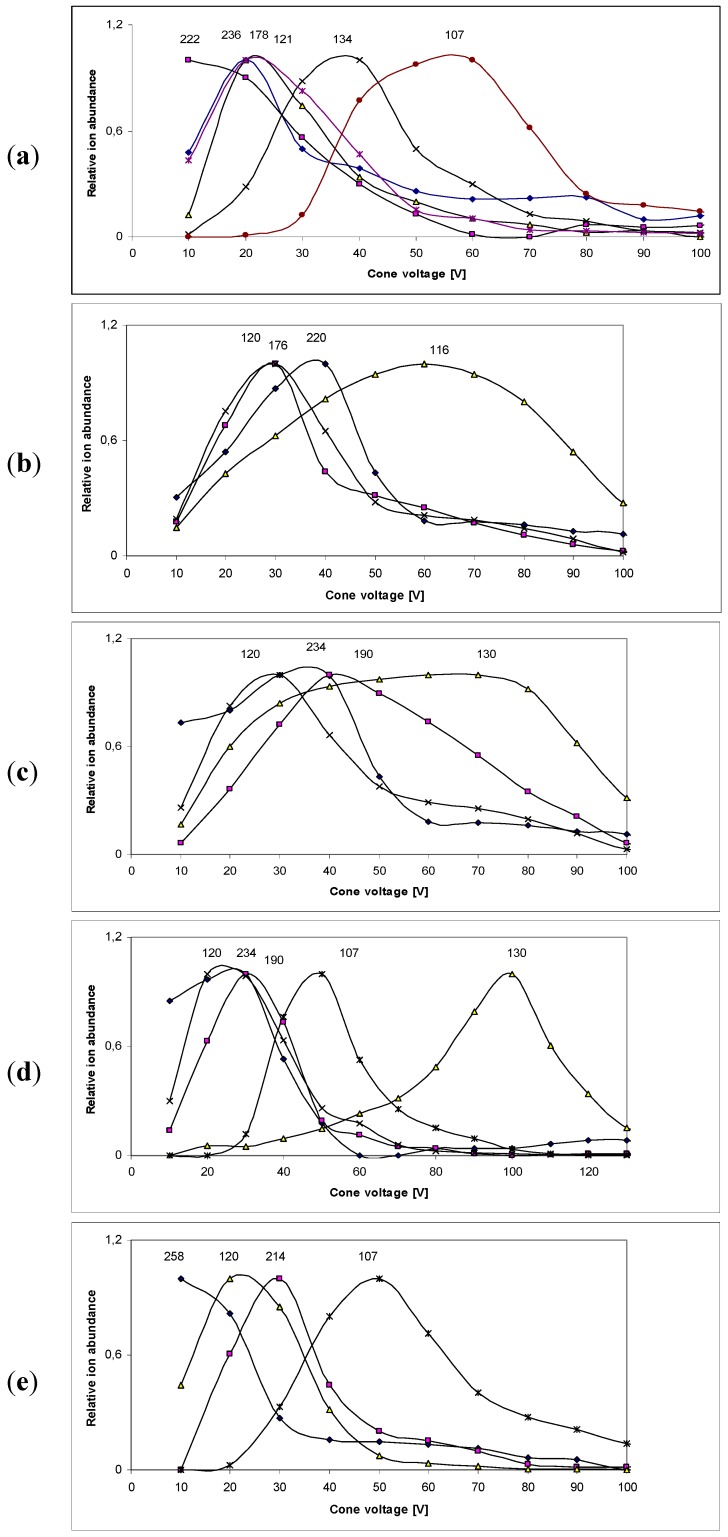
The correlations of relative ion abundance *vs.* cone voltage for compounds: **1** (**a**); compound **2** (**b**); compound **3** (**c**); compounds **4** (**d**) and compound **5** (**e**).

The negative ESIMS spectra were recorded as a function of cone voltage in 10–180 V range. The correlations of relative ion abundance *vs.* cone voltage for compounds **1**–**5** are collected in Figure 5.

The dependence of ion abundance on cone voltage is determined by the ions’ stability in the gas phase and this stability is responsible for the relative intensity of the *m/z* signals at a constant cone voltage. The stabilities of both precursor and fragment ions depend on the structure of the amino acid Schiff bases. Throughout the cone voltage range tested, a high instability of negative ions in the gas phase was noted.

## 3. Experimental

### General

**Scheme 2:**
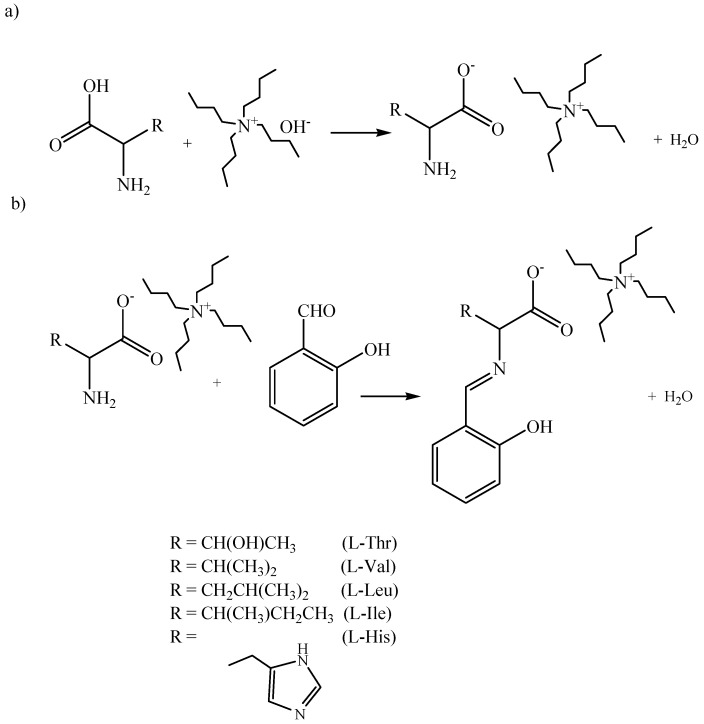
Synthesis of amino acid ionic liquid supported Schiff bases.

Tetrabutylammonium salts of amino acids were prepared by the reaction of a slight molar excess of amino acid dissolved in water and 40% aqueous tetrabutylamonium hydroxide solution (Scheme 2a). After the reaction, water was removed under reduced pressure [34]. The intermediate product was dried in a vacuum oven at 60 °C under a pressure of 2 mbar for 24 h. Amino acid ionic liquid-supported Schiff bases were synthesized by condensation of tetrabutylammonium salts of amino acids with salicylaldehyde according to the procedure described elsewhere (Scheme 2b) [19]. The reaction was carried out in absolute ethanol. After completion of the reaction, the solvents were removed under reduced pressure. The product in the form of yellow oil was dried under reduced pressure. Compound 5 was obtained in the form of yellow needles (melting point 74 °C). All compounds studied were stable in DMSO, ethanol and chloroform, although in chloroform solutions after *ca.* 1h some symptoms of compound **5** decomposition were observed.

The ^1^H and ^13^C-NMR spectra were recorded on a Bruker DPX-400 spectrometer operating at 100.62 MHz (^13^C) and 400.13 MHz (^1^H), respectively. Typical spectral parameters were used for ^13^C-NMR: spectral width 24 kHz, number of data points 65.5 K, 1.46 Hz per point digital resolution, acquisition time 1.37 s, relaxation delay 1 s, pulse width 9.2 μs, number of scans 1,000–8,000; for ^1^H-NMR: spectral width 12 kHz, number of data points 65.5 K, 0.488 Hz per point digital resolution, acquisition time 4.09 s, relaxation delay 1 s, pulse width 7.8 μs, number of scans 16. The chemical shifts were referred to TMS as internal standard. Typical concentration of the samples was 0.1 M. The temperature was maintained and measured with Eurotherm BV-T 2000 to an accuracy of 1 K. Deuteration of the compounds was achieved by dissolving the sample in CH_3_OD followed by evaporation under reduced pressure.

The diffuse reflectance FT IR spectra were measured on Nicolet 380 Thermo Electron Corporation on KBr pellets. UV**-**Vis absorption spectra were measured using a Spectroquant^®^ Pharo 300 Spectrophotometer from Merck to the accuracy of ±1 nm in a 10 mm quartz cell. The concentration of solutions for all compounds was in range 10^−4^–10^−5^. The absolute ethanol was purchased from EUROCHEM, spectral grade chloroform from POCh.

NMR, FT-IR and UV-Vis spectra are available as supporting information.

The specific rotation of new amino acids ionic liquids-supported Schiff bases was investigated on Polarymetr Autopol IV from Rudolph Research Analytical. Mass spectra (MS) were recorded on ZQ Waters/Micromass Mass Spectrometer (Manchester, UK) with quadrupole analyser with the following parameters used: source potential ESI on capillaries: 3 kV; voltage on focal pla: 0.5 V; voltage on extract: 4V; the cone voltage (cv): 30 V, ion fragmentation was examined with 10–180 V (*cv*); source temperature: 120 °C; evaporation temperature: 300 °C; nitrogen was used as a spraying and drying gas at the flow rate of 80 and 300 L h^−1^.

ESI mass spectra of negative and positive ions of compounds 1–6 were recorded in MCA mode (Multi Channel Acquisition) in *m/z* = 100–1,000 interval. The typical spectrum obtained was the average of 10 scans with 0.6 s time interval. The solutions studied were introduced to the ionization source (at the flow rate 40 μL min^−1^) through a Harvard Scientific pump. All the solutions subjected to ESI MS were prepared in methanol.

## 4. Conclusions

Spectroscopic studies have shown that the tetrabutylammonium salts of amino acid Schiff base derivatives of salicylaldehydes **1**–**4** exist in tautomeric equilibrium. The position of proton transfer equilibrium was shifted towards the NH-form at a mole fraction above 0.5 for the derivatives of l-valine, l-leucine and l-isoleucine at low temperatures in a chloroform solution. For the l-threonine derivative **5** the equilibrium was shifted towards the OH-form, while the l-histidine derivative existed in chloroform and DMSO solutions exclusively in the OH-form. It was confirmed that the proton transferred form was stabilized by a bifurcated intramolecular hydrogen bond. The presence of the imidazole ring weakens the interactions between the COO- and NH groups, which stabilizes the proton transferred form.

## Supplementary Materials

Supplementary materials can be accessed at: http://www.mdpi.com/1420-3049/18/5/4986/s1.
