# Supplementary material for: Spectroscopic Studies of Amino Acid Ionic Liquid-Supported Schiff Bases
Source: Molecules. 2013 Apr 29;18(5):4986–5004. doi: 10.3390/molecules18054986 (PMC6269710; doi:10.3390/molecules18054986)

**Supporting Information (NMR, IR, UV-Vis spectra)**

**(**1) Sal-L-Thr CDCl_3_ 295K

**(**1) Sal-L-Thr CDCl_3_ 230K

**(**1) Sal-L-Thr DMSO 295K

**(**2) Sal-L-Val CDCl_3_ 295K

**(**2) Sal-L-Val CDCl_3_ 230K

**(**2) Sal-L-Val DMSO 295K

**(**3) Sal-L-Leu CDCl_3_ 295K

Sal-L-Leu CDCl_3_ 230K

**(**3) Sal-L-Leu DMSO 295K

**(**4) Sal-L-Ile CDCl_3_ 295K

**(**4) Sal-L-Ile CDCl_3_ 230K

**(**4) Sal-L-Ile DMSO 295

**(**5) Sal-L-His CDCl_3_ 295K

**(**4) Sal-L-Ile CDCl_3_ 230K

**(**4) Sal-L-Ile DMSO 295K

**(**1) Sal-L-Thr


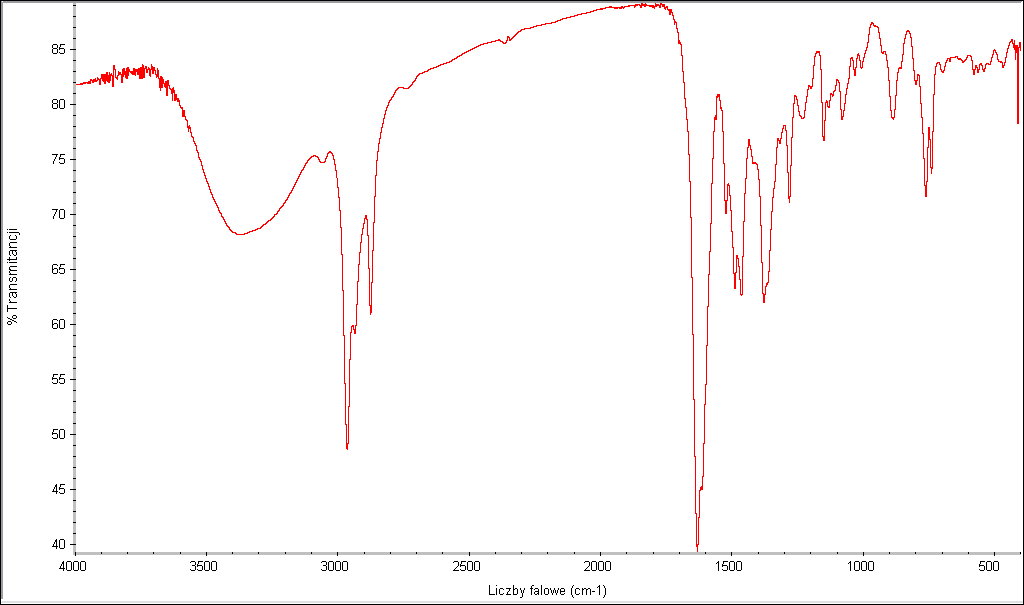


**(**2) Sal-L-Val


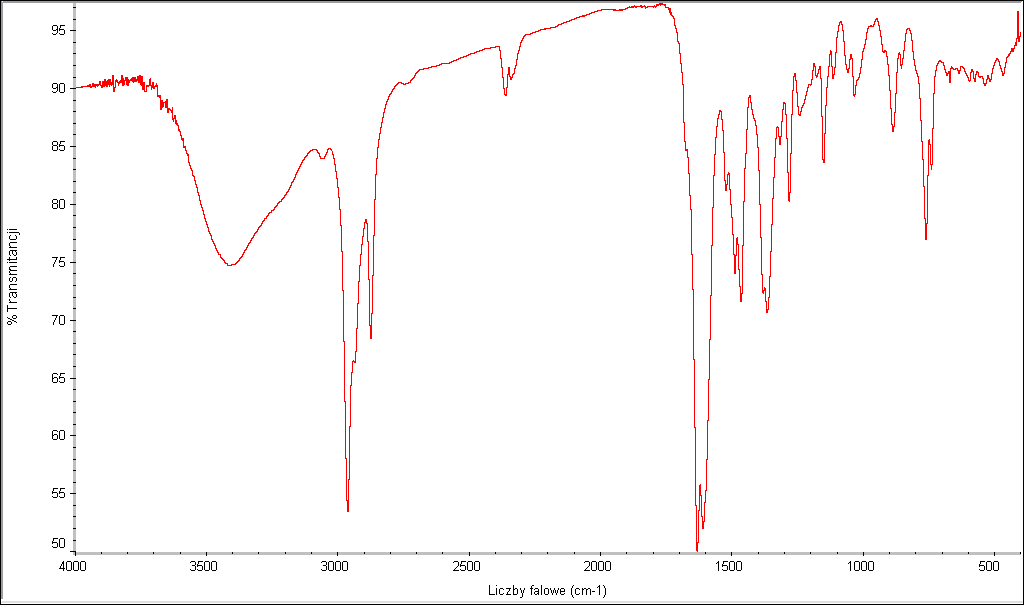


**(**3) Sal-L-Leu


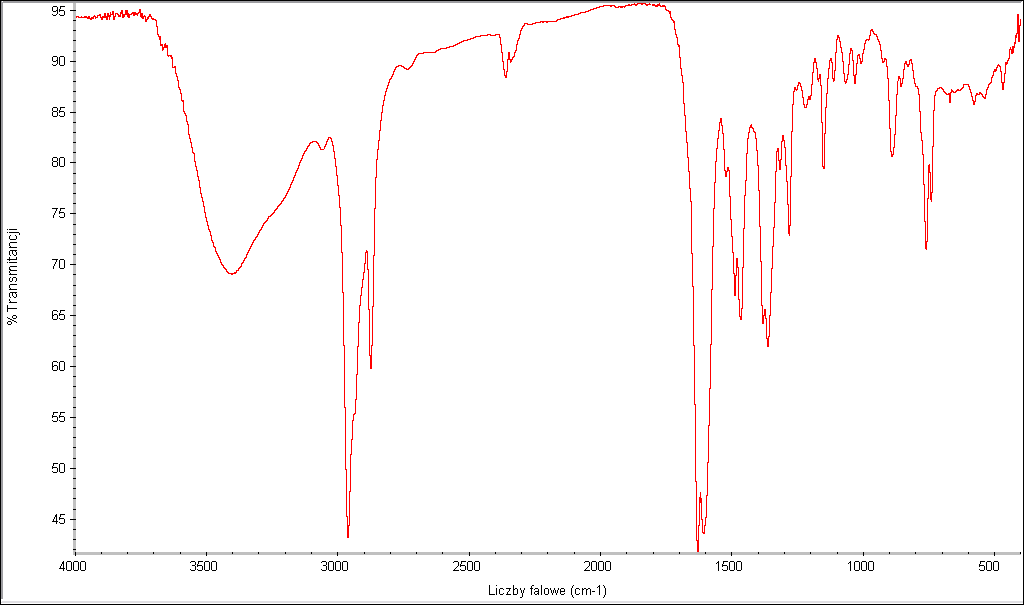


**(**4) Sal-L-Ile


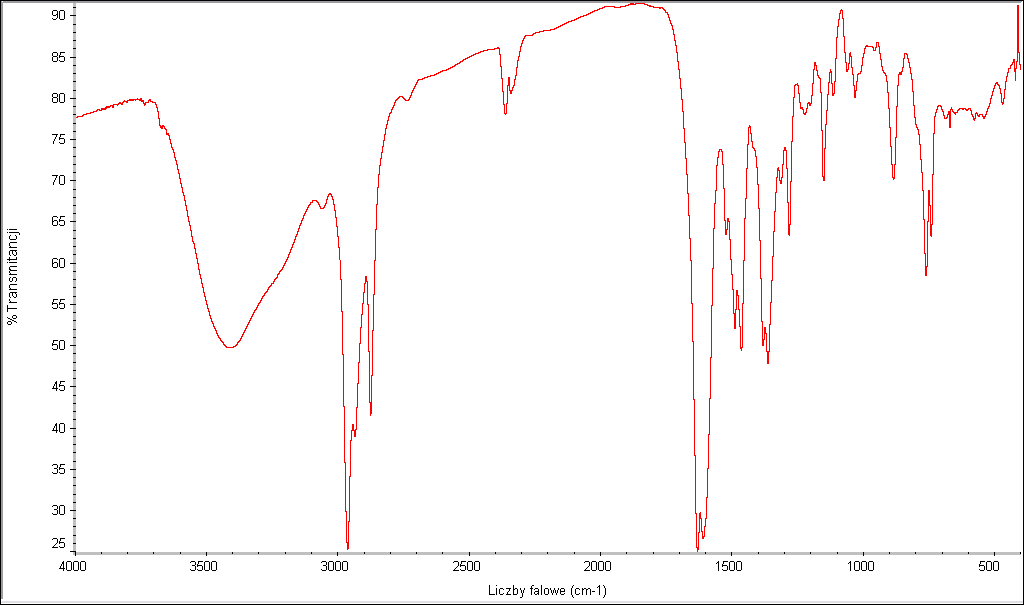


**(**5) Sal-L-His


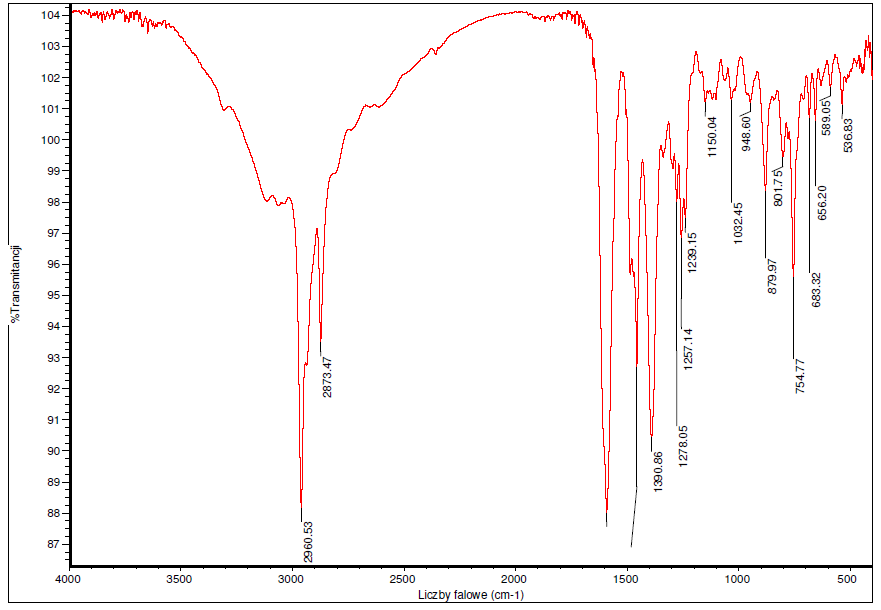


**(**1) Sal-L-Thr CHCl_3_


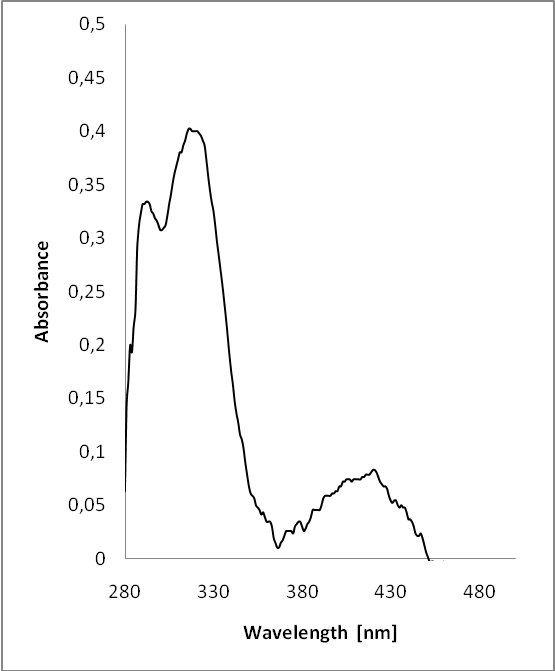


**(**1) Sal-L-Thr EtOH


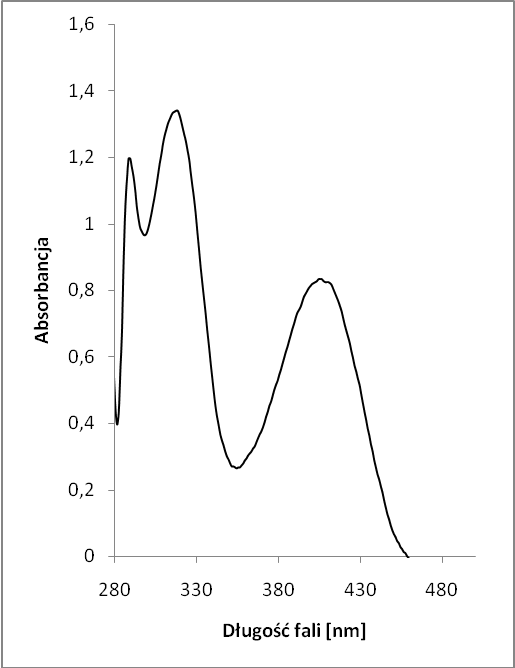


**(**2) Sal-L-Val CHCl_3_


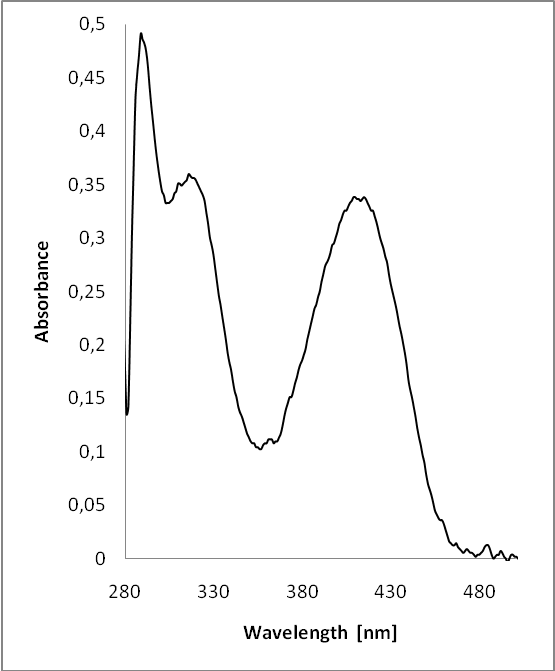


**(**2) Sal-L-Val EtOH


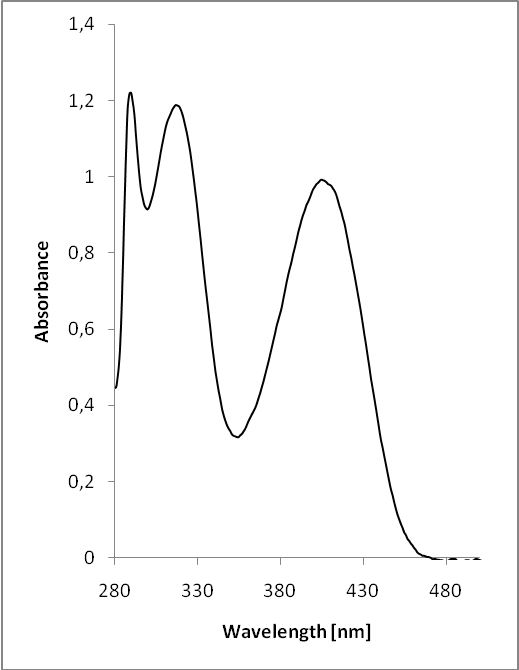


**(**3) Sal-L-Leu CHCl_3_


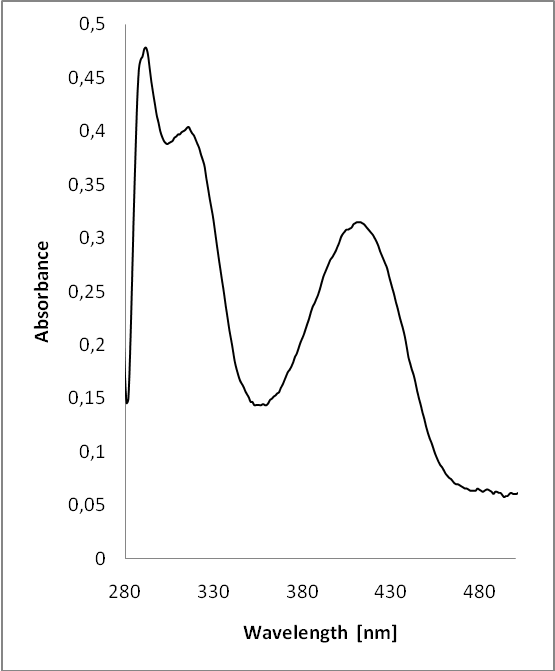


**(**3) Sal-L-Leu EtOH


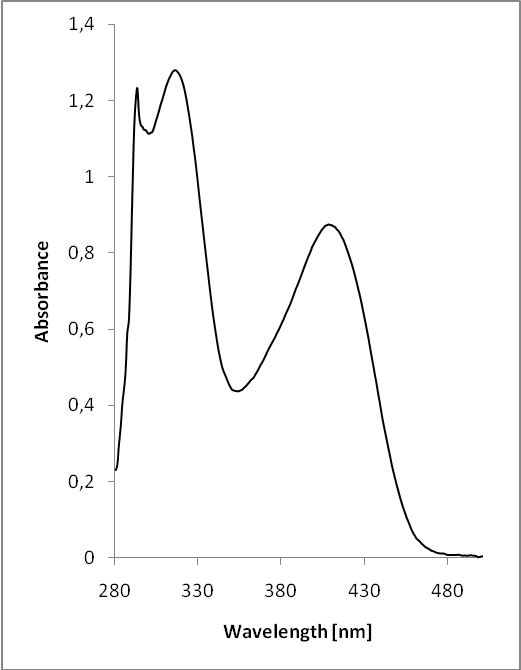


**(**4) Sal-L-Ile CHCl_3_


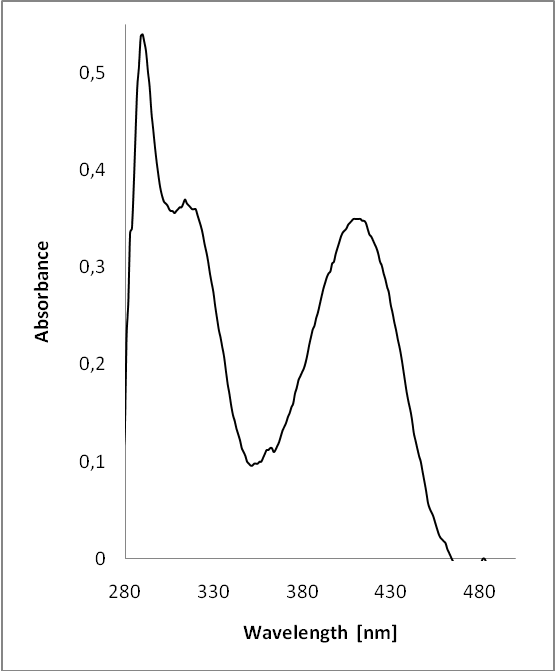


**(**4) Sal-L-Ile EtOH


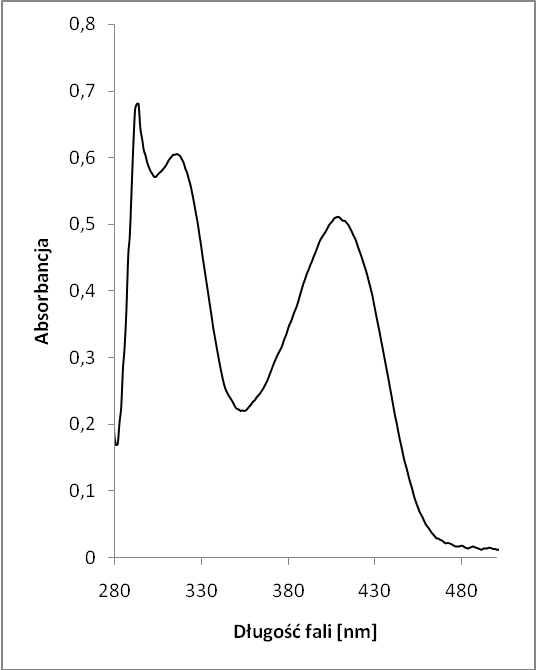


**(**5) Sal-L-His CHCl_3_


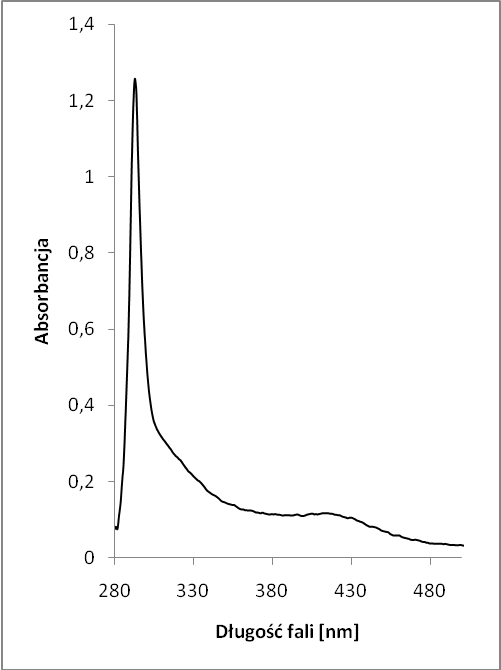


**(**5) Sal-L-His CHCl_3_


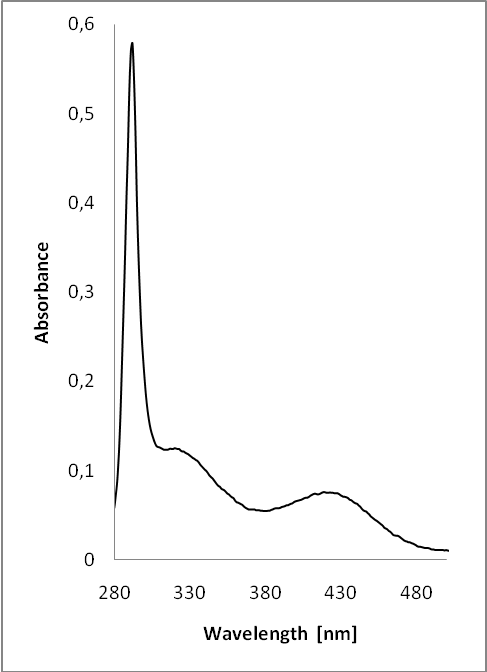

Supplement: Supplementary file 1 [file molecules-18-04986-s001.doc]
